# Exploration of the One-Bead One-Compound Methodology for the Design of Prolyl Oligopeptidase Substrates

**DOI:** 10.1371/journal.pone.0006222

**Published:** 2009-07-13

**Authors:** Gemma Comellas, Zusanna Kaczmarska, Teresa Tarragó, Meritxell Teixidó, Ernest Giralt

**Affiliations:** 1 Institut de Recerca Biomèdica (IRB Barcelona), Parc Científic de Barcelona, Barcelona, Spain; 2 Department of Organic Chemistry, Universitat de Barcelona, Barcelona, Spain; University of Helsinki, Finland

## Abstract

Here we describe the design, synthesis and evaluation of the first solid-phase substrates for prolyl oligopeptidase (POP), a cytosolic serine peptidase associated with schizophrenia, bipolar affective disorder and related neuropsychiatric disorders. This study seeks to contribute to the future design of a one-bead one-compound (OBOC) peptide library of POP substrates, based on an intramolecular energy transfer substrate. Unexpectedly, the enzymatic evaluation of the substrates attached on solid-phase by means of the HMBA linker were cleaved through the ester bond, thereby suggesting an unknown esterase activity of POP, in addition to its known peptidase activity. By performing multiple activity assays, we have confirmed the esterase activity of this enzyme and its capacity to process the substrates on solid-phase. Finally, we tested a new linker, compatible with both the solid-phase peptide-synthesis used and the enzymatic assay, for application in the future design of an OBOC library.

## Introduction

During recent decades, recognition of the importance of neuropeptides in the correct functioning of the central nervous system (CNS) has led to the evaluation of various proteases in the human brain as therapeutic targets. Among these, prolyl oligopeptidase (POP, EC 3.4.21.26) is of particular relevance because of its association with schizophrenia, bipolar affective disorder and related neuropsychiatric disorders. In 1995, Maes and collaborators detected a higher activity of the POP enzyme in patients suffering from these illnesses than in healthy subjects [1]. For years, several research groups have focused on the development of inhibitors of the porcine POP enzyme. However, no specific inhibitor is currently in clinical use. Consequently, intense research effort has been devoted to revealing the basic structure, ligand binding and kinetic properties of POP [2], [3].

In 1998, the three-dimensional structure of porcine POP was published; it consists of two domains: the peptidase domain and the non-catalytic structural domain [4]. In 2005, the cloning of POP from human brain RNA was achieved, and a homology model of the human POP based on porcine POP structure was designed [5]. Since then and using the expressed human brain POP, new inhibitors have been reported, such as berberine [6], [7]. These results provide an excellent starting point for the design of a library of substrates in order to elucidate the connection between the substrates and the activity of the enzyme for the future design of efficient POP inhibitors.

During the last decade, combinatorial libraries have received much attention from the pharmaceutical industry because they can significantly facilitate the drug discovery process; in particular, interest in one-bead one-compound (OBOC) peptide libraries has recently increased.

Here we present the results of the first attempt to design an OBOC peptide library for the POP enzyme, based on an intramolecular energy transfer substrate (IFETS) published by Fulop *et al*. [8]. After being processed by the enzyme, the compounds lead to the separation of the intermolecular donor-acceptor pair, thus allowing an increase in the fluorescence proportionally to the amount of hydrolyzed peptide. The active substrates will then be physically isolated for analysis and future structural characterization.

The principle of an OBOC combinatorial library is the synthesis of many compounds in solid-phase by a “Mix-Split” approach [9]. This methodology presents many copies of the same compound on a single bead. Each bead contains only one compound and many of these compound beads are synthesized simultaneously. In order to use this kind of library for a specific target, the parameters must be established in order to ensure a good screening methodology, which includes: the selection of the model compound, with a fluorophore and a quencher; a proper linker; and the selection of a resin to immobilize the compound and ensure the proper enzymatic assay, among other parameters. Here we describe the results we obtained during the fine-tuning of an OBOC peptide library for POP, based on a substrate described by Fulop *et al*. [8]. These results provide the first step for a future OBOC library.

## Results and Discussion

### Model substrate synthesis

The original porcine POP brain substrate described by Fulop *et al*. [8] and the two expected fragments from the enzyme processing were prepared using solid-phase peptide synthesis (SPPS). After optimizing the synthetic methodology, the compounds were obtained with purity higher than 90% and were characterized by HPLC-UV, HPLC-MS and MALDI-TOF MS.

### Enzymatic evaluation of the model substrate in solution

**Figure 1:**
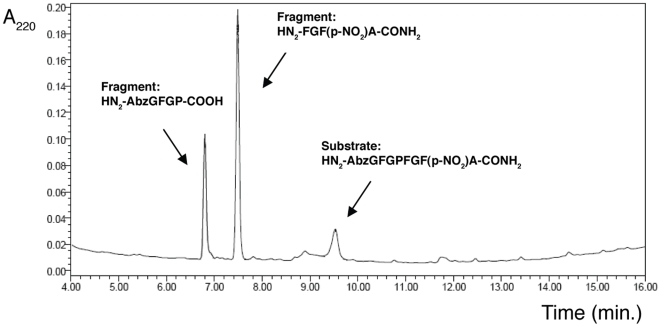
RP-HPLC Chromatogram of the enzymatic evaluation of the model substrate. Conditions: lineal gradient of 0–100% MeCN (0.036% TFA) in H_2_O (0.045% TFA) in 15 min. Flux of 1 mL/min, in a symmetry column C_18_ (4.6 mm×150 mm), Waters, 100 Å, 5 µm, detection at 220 nm.

Fulop *et at*. reported that the model substrate was a substrate of the porcine POP enzyme [8]. In the present study, the substrate was incubated in 100 mM Na^+^/K^+^ phosphate buffer pH 8.0 with diverse concentrations of the human brain POP and the model substrate for 1 h at 37°C. The reaction was stopped by heating at 95°C for 5 min to denaturalize the enzyme and then evaluated by HPLC-UV (Figure 1), MALDI-TOF spectrometry and fluorescence. This experiment demonstrated that the compound is also a human brain POP substrate.

After analyzing the non-autofluorescence of POP at the working range of wavelengths and characterizing the fluorescence of the two fragments, we confirmed that quenching occurs via a collision mechanism. This observation must be taken in consideration during the design of a future OBOC peptide library [10] as the interaction between the two groups occurs at a shorter distance range.

### Design of the solid-phase analogs

Peptide substrates, such as the model compound, are commonly used for determining the activity of a proteolytic enzyme in solution. However, when the substrate is attached to a solid support, additional concerns must be considered: the interference of the solid support with the substrate hydrolysis to ensure sufficient space for the enzyme reaction (usually solved with the presence of spacer molecules to take the reaction site further away from the solid support); the need for a specific free terminal end of the peptide to reach the active site and to be hydrolyzed by the enzyme (related to the enzymatic mechanism); and the substrate recognition effect produced by the sequence changes in order to obtain a fluorescent bead after the hydrolysis.

**Figure 2:**
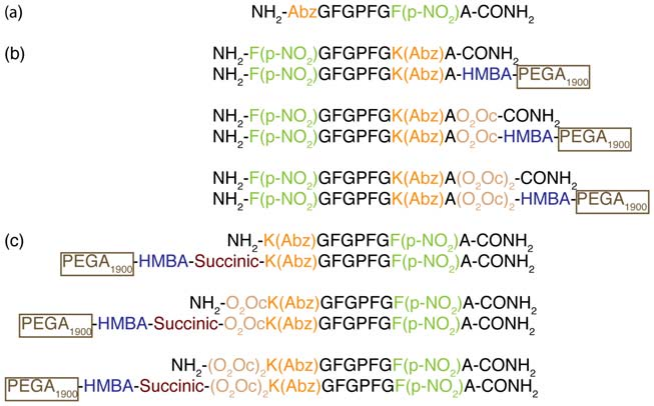
Representation of (a) the model substrate, (b) the *C*-terminal-anchored analogs and control peptides, (c) the *N*-terminal-anchored analogs and control peptides.

To design the solid-phase analogs, the structure of the substrate situated at the active site of the POP enzyme, obtained by Fülöp and coworkers, was considered [8]. The designed analogs are shown in Figure 2. On the basis of the homology model [5], we considered necessary the presence of a spacer molecule between the substrate and the linker necessary to allow the anchored peptide to enter the active site. A hydrophilic spacer consisting of a polyethylene glycol, the 8-amino-3,6-dioxaoctanoic acid (abbreviated as O_2_Oc), was selected to permit accessibility and compatibility with the enzymatic assay. To identify the characteristics required for a solid-phase anchored peptide to be a POP substrate, we designed several peptides.

These peptides were divided into two groups depending on the peptide end attached to the resin. A number of derivates were attached to the solid support by the *C*-terminal end, while the others were attached by the *N*-terminal end. In order to design an OBOC peptide library, the positions of the fluorophore and the quencher moieties were changed in the *C*-terminal-anchored peptides to obtain a fluorescent bead after the hydrolysis of the substrate by the POP enzyme (Figure 2). The linker on the polymer was also selected to allow easy peptide sequence analysis after the enzymatic evaluation compatible with the SPPS. Moreover, control peptides (not anchored to the resin) for each anchored analog were also prepared to ensure that changes to the peptides were not affecting their recognition by the POP enzyme.

### Selection of the resin

**Figure 3:**
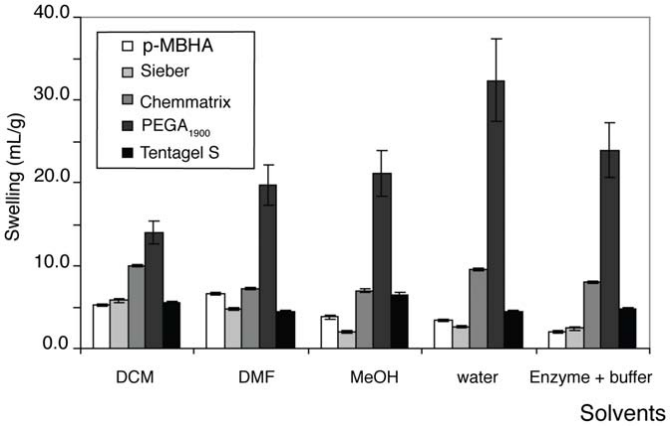
Swelling of the resins in five different solvents in mL/gr.

To select the most appropriate resin for the synthesis and the following enzymatic assay, three PEG-based resins (Tentagel Standard, Aminomethyl Chemmatrix and PEGA_1900_) and two control polystyrene resins (Sieber and *p*-MBHA) were selected. First of all, we examined the effect of the employed solvents by measuring the swelling of the selected resins (figure 3). These measurements showed that PEGA_1900_ had better swelling properties than Tentagel S and Chemmatrix resins. In contrast to polystyrene resins, PEGA_1900_ showed similar swelling in organic and aqueous systems, especially when we compare the DMF where the synthesis will be carried out and the buffer with enzyme in which the peptide will be assayed. PEGA_1900_ resin has the best swelling properties for the purposes of a successful synthesis and enzymatic evaluation.

Another key factor for the design of an OBOC peptide library is bead homogeneity. In the future library, each bead will contain a distinct compound and its fluorescence should depend only on the nature of the compound, not on the amount of compound on the bead. Optical microscopy images of the resin swelled in buffer with the POP enzyme were taken and the beads were characterized in terms of size and size distribution. These measurements showed that Tentagel S and PEGA_1900_ had a more homogenous bead size than Aminomethyl-Chemmatrix. Finally, the auto-fluorescence of resins was measured under several light sources. No resin showed high fluorescence under the working conditions; only a very small reminiscent auto-fluorescence was detected.

Another crucial feature of the set-up is the capacity of the enzyme to penetrate the support beads. Previous studies showed that Tentagel S presents limitations in aqueous solution, where enzymatic catalysis takes place, as a result of the presence of DVB as cross-linking agent. In contrast, peptides attached to PEGA_1900_ are processed by the enzyme in near quantitative yield [11], [12]. The evaluation of a model compound with an enzyme of the same molecular weight as POP, the penicillin acylase, presented no enzymatic cleavage when Tentagel S was used, while partial cleavage was observed using PEGA_1900._ These observations demonstrated that PEGA_1900_ shows better performance in terms of bio-compatibility than Tentagel S [13].

PEGA resin can take several forms depending on the chain length, which affects enzyme accessibility. There is evidence that PEGA beads restrict the penetration of enzyme molecules, particularly larger ones. Previous studies report that PEGA_4000_ and PEGA_1900_ can be used for enzymes with a molecular weight of up to 120KDa and 60KDa, respectively [14]_._ Since the enzyme pores limit its diffusion to the core of the particle, enzyme-mediated reactions occur only at the surface [15], [16]. If the reaction occurs only at the surface, some entire peptide will remain in the core of the bead. If this is the case, it could be an interesting advantage in our case, since, by evaluating the entire peptide in the positive bead, it will allow us to change more residues of the peptide in the future OBOC peptide library located in the fragment expected to be released. We therefore chose to use PEGA_1900_ for our purposes.

### Synthesis of the analogs

**Figure 4:**
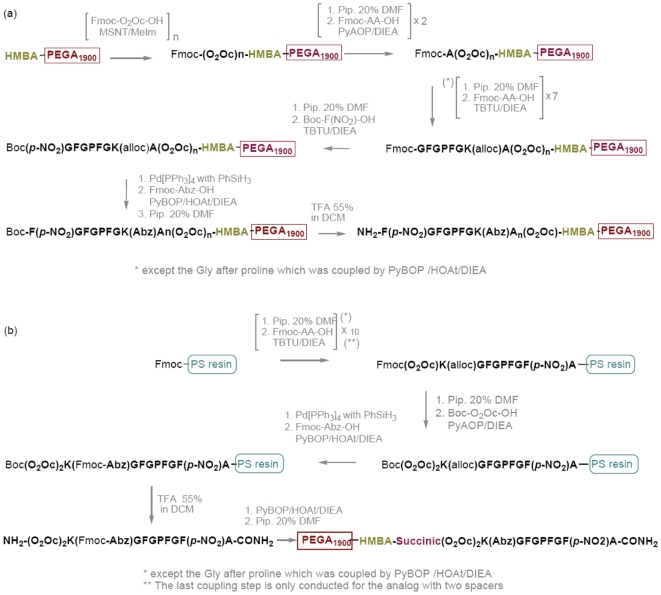
General representation for the synthesis of the anchored analogs. (a) *C-*terminal analogs and (b) *N*-terminal analogs.

The solid-phase analogs were synthesized by SPPS. To prevent the premature and undesired removal of the Fmoc-protecting group of the ^α^ *N*-amino acids by the ε–amino of the Lys [17], an orthogonal protection scheme of the ^α^ *N*-amino acid and the ^ε^ *N*-Lysine was used for the synthesis of the analogs. The orthogonal protection scheme included Fmoc/^t^Bu chemistry and allyloxycarbonyl (Alloc) protection and also Fmoc/^t^Bu chemistry and Boc protection. Figure 4 presents a general scheme of the analog syntheses.

The amino group was modified by attaching an appropriate linker, the 4-hydroxymethylbenzoic acid (HMBA) linker, which is compatible with Fmoc/^t^Bu chemistry and Boc and Alloc protection and described to be stable in activity assays [16]. In the synthesis of the *N*-terminal-anchored peptides, succinic anhydride was coupled after the HMBA linker, thereby allowing the re-anchoring of the *N*-terminal analogs and possible cleavage in order to identify the compounds.

The *C*-terminal-anchored analogs were directly synthesized in PEGA_1900_ resin containing the HMBA linker, while the control peptides of the *C*-terminal-anchored analogs were synthesized in polystyrene resin. Usually SPPS proceeds from the *C*-terminal to *N*-terminal, because previous attempts in the opposite direction encountered inherent problems that limited its general application [18]–[22]. Consequently, the *N*-terminal-anchored peptides were synthesized by using polystyrene, cleaved and re-anchored by the *N*-terminal to the PEGA _1900_ resin. If the future library is designed based on *N*-terminal peptides, then a similar synthetic strategy to Yao *et al.* would be used to obtain the free carboxyl end peptides [18].

**Figure 5:**
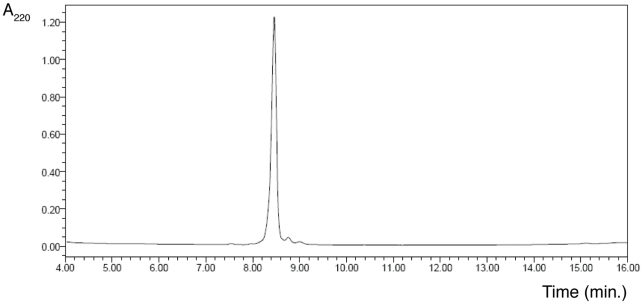
RP-HPLC Chromatogram of an analog peptide (two spacers) before being anchored by the *N*-terminal side. Conditions: lineal gradient of 0–100% MeCN (0.036% TFA) in H_2_O (0.045% TFA) during 15 min. Flux of 1 mL/min, in a symmetry column C_18_ (4.6 mm×150 mm), Waters, 100 Å, 5 µm, detection at 220 nm.

After optimizing the methodology, the peptides were obtained in good purity (no purification was required) and yield. For instance, Figure 5 shows the analog synthetic methodology containing two spacers before being anchored by the *N*-terminal side.

**Figure 6:**
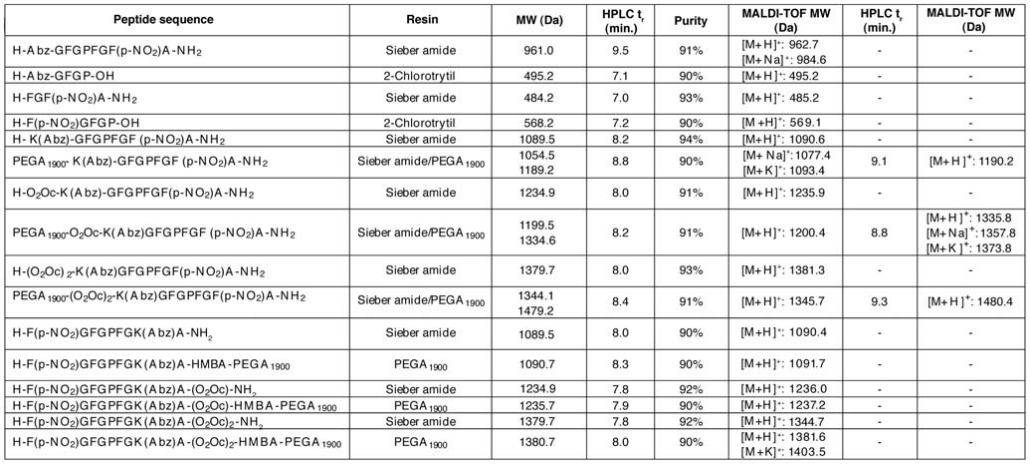
Purities and HPLC elution times in a lineal gradient 0–100% MeCN-H_2_0 (15 min), and MALDI-TOF MW of the synthesized peptides. Control cleavages are also reported for the peptides synthesized or reanchored into PEGA_1900_ resin.

After the synthesis, control cleavages of all the compounds were analyzed by HPLC-UV and MALDI-TOF. All compounds were obtained in good purity (Figure 6).

**Figure 7:**
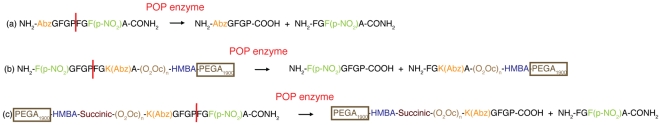
General scheme of the peptides processed by the POP enzyme and the fragments generated. (a) Model substrate (b) *C*-terminal-anchored peptides (c) *N*-terminal-anchored peptides.

If the POP enzyme processes the anchored analogs, only one of the two fragments will be released, thereby allowing its detection in the solution by HPLC-UV. The fragments generated by the three analogs in each group, the *N*-terminal or the *C*-terminal groups, were the same as shown in Figure 7. And the fragment generated from the *N*-terminal analogs will be the same as one of the fragments generated by the model substrate. Consequently, only the fragment generated by the *C*-terminal analogs was synthesized.

### Enzymatic evaluation of the analogs

All the control peptides (peptides in solution) were incubated under the same conditions and following the same methodology as the model substrate; 90 µM of the control peptide with a range of concentrations of the POP enzyme in Na^+^/K^+^ phosphate buffer pH 8.0 (100 mM) during 15 min, 30 min, 1 h and 3 h at 37°C.

Although the peptide sequences presented modifications compared to the model substrate, analyses by HPLC-UV and HPLC-MS confirmed that the control peptides were also POP substrates. This result indicated that the sequence modifications did not affect the recognition of the peptides as POP substrates.

**Figure 8:**
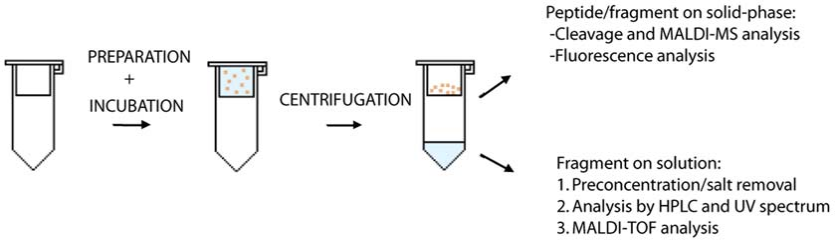
Scheme of the activity assay preparation and analysis of the anchored analogs.

The peptides attached to the solid support, PEGA_1900_ resin, were incubated with the POP enzyme by using Sigma Corning Spin-X centrifuge tube filters, as shown in Figure 8. After conducting the activity assay, the solution and the beads were separeated by centrifugation. After a treatment to remove the POP enzyme, desalt and pre-concentrate the peptide, the filtrate was analyzed by HPLC-UV and MALDI-TOF or HPLC-MS. Finally, the peptides attached on the bead were cleaved from the resin and analyzed by MALDI-TOF or directly analyzed under a fluorescence microscope.

The anchored analogs at a concentration of 300 µM were incubated with a range of human POP concentrations and times (usually longer incubations than the control peptides) in Na^+^/K^+^ phosphate buffer pH 8.0 (100 mM) at 37°C. If the enzyme was processed the anchored analogs, this methodology would permit the detection of the fragment containing the fluorophore in the bead and the fragment containing the quencher in the solution. Unexpectedly, the solution analysis detected the presence of both fragments, thereby suggesting the processing of the substrates by POP but also cleavage from the solid-phase. But the question raised was whether the compounds were processed by the enzyme and then released from the solid-phase or whether they were cleaved and then processed by the enzyme.

If the POP had processed the compounds attached on solid-phase, then the beads could still contain some attached fragment. After incubating the substrates on solid-phase with POP, several beads were placed in a MALDI plate and treated with NH_3_ vapour and analyzed by MALDI-TOF. Another fraction of beads were cleaved with NaOH and analyzed by HPLC-UV and HLPC-MS. The fragment was not detected. Therefore, we could not confirm whether the POP was processing the fragments on solid-phase or in solution.

To analyze whether the medium was cleaving the peptides from the resin, the same assays were conducted at different pHs (6, 7 and 8) and incubation times with and without POP. No cleavage of the peptide was observed in the absence of the enzyme, thereby demonstrating that the media was not cleaving the peptides from the solid support.

To determine whether something present in the POP media was cleaving the peptide, POP pre-incubated with the ZPP (z-prolil prolinal), a potent POP inhibitor of the enzyme, was assayed. No cleavage in the presence of the inhibitor was detected. The incubation of the solid-phase substrate and the fragment generated by POP in the presence of the entire peptide was conducted and no cleavage was observed (to discard the self-cleavage produced by the fragments generated). These results suggested that POP acted as an esterase, in addition to its known peptidase activity.

**Figure 9:**
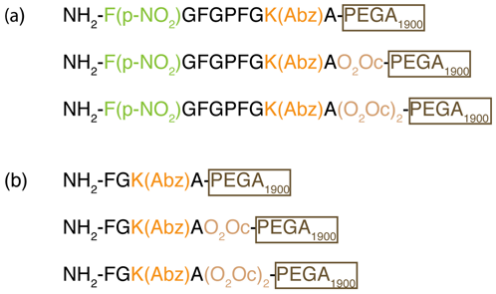
Representation of the peptides used to confirm the esterase activity and capacity to process on solid-phase. (a) *C*-terminal-anchored analogs directly bounded on solid-phase. (b) *C*-terminal control peptides for the fluorescence assay directly bounded on solid-phase.

To confirm the esterase activity of POP and its capacity to process the analog peptides on solid-phase, we synthesized and evaluated new solid-phase peptides. The new derivates were *C*-terminal-anchored analogs directly bound on solid-phase (without a linker), as shown in Figure 9a. These derivates were not intended for the design of a future library, as they could not be cleaved for evaluation, but were used only to test this hypothesis.

**Figure 10:**
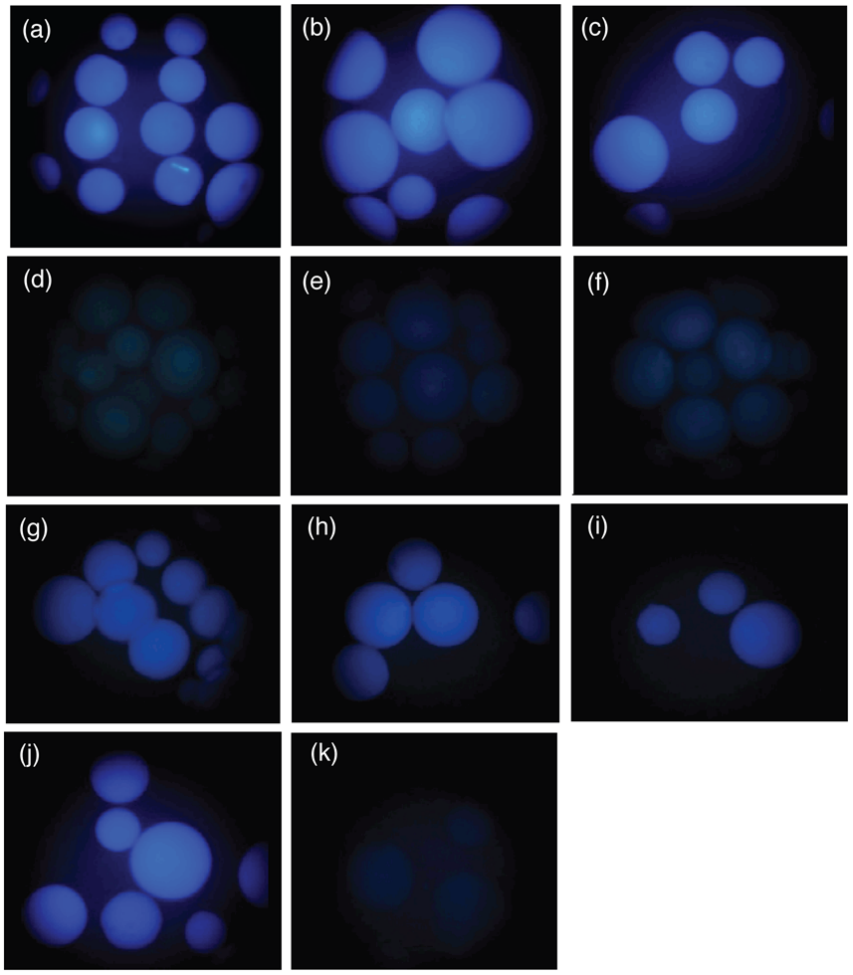
Fluorescence microscope images of the peptides shown in figure 8. The images were taken at the excitation wavelength range of 360/40 nm and emission wavelength range of 420/40 nm. Peptides not assayed with the POP enzyme: (a) NH_2_FGK(Abz)A-PEGA_1900_ (b) NH_2_FGK(Abz)O_2_Oc-PEGA_1900_ (c) NH_2_FGK(Abz)(O_2_Oc)_2_-PEGA_1900_ (d) NH_2_F(*p*-NO_2_)GFGPFGK(Abz)A-PEGA_1900_ (e) NH_2_F(*p*-NO_2_)GFGPFGK(Abz)O_2_Oc-PEGA_1900_ (f) NH_2_F(*p*-NO_2_)GFGPFGK(Abz)(O_2_Oc)_2_-PEGA_1900._ Images of the peptides (d) to (f) after the assay with the POP enzyme are shown in Figures (g) to (i). Fluorescence controls are (j) NH_2-_Abz-PEGA_1900_ (positive control) and (k) PEGA_1900_ (negative control).

After the enzymatic assay, the solution was analyzed by HPLC-UV and MALDI-TOF analysis (Figure 8) showing the presence of the only expected fragment. These results were also supported by the bead fluorescence analysis (Figure 10). The fluorescence of the fluorescent fragment synthesized directly on solid-phase (Figure 9b) was compared with the fluorescence of the beads after the incubation with the enzyme and the beads without incubation (see Figure 10). These experiments confirmed the esterase activity of POP and also corroborated the capacity of POP to process the substrates bound on PEGA_1900_ resin by the *C*-terminal end. Moreover, our results demonstrated that POP can process the substrates on solid-phase without the need of spacers, thereby simplifying the future design of an OBOC peptide library and also showing the capacity of POP to penetrate the PEGA_1900_ resin.

**Figure 11:**
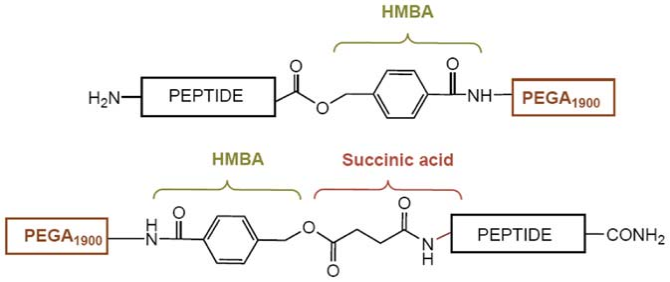
Representation of the linker system used in the (a) *C*-terminal analogs (b) *N*-terminal analogs.

**Figure 12:**
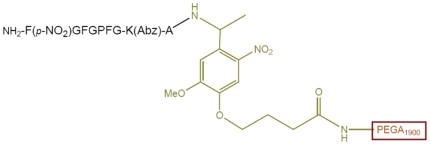
Representation of the peptide bound to PEGA_1900_ resin using the PLL linker (drawn in green).

These results confirmed the incompatibility of the HMBA linker in our system with the POP enzymatic assay. Consequently, a distinct linker will be necessary for the future design of an OBOC peptide library (Figure 11). We present the initial results on the use of the photolabile linker (PLL), by using the Fmoc-4-[4-(1-aminoethyl)-2-methoxy-5-nitrophenoxy]butyric acid (Fmoc-photo labile linker; Fmoc-PLL) [23]. The linker, which presents no ester bonds, was tested with the peptide shown in Figure 12.

The peptide was based on the *C*-terminal side analog using the PLL linker to bond to the resin (no spacers were used). After the enzymatic evaluation, only the expected fragment was present in the solution, as shown by HPLC-UV and MALDI-TOF analyses. These results confirmed the synthetic and enzymatic biocompatibility of the new linker.

### Conclusions

We have demonstrated that the porcine POP substrate described by Fulop *et al.* is also a human POP substrate. We have characterized this porcine POP substrate for the design of an OBOC peptide library for the human POP enzyme; this represents the first attempt to immobilize a POP enzyme substrate in solid-phase. After the design, synthesis and evaluation of the analogs of this substrate, our results confirmed that the changes in the sequences did not interfere in the recognition by the enzyme. However, the solid-phase analogs were cleaved from the solid support during the enzymatic evaluation. Our enzymatic analyses confirmed that the cleavage was not caused by the media, the pH or the generated fragments, thereby suggesting that the POP enzyme also acts as an esterase. By directly attaching the substrate to the resin, we proved the esterase activity of the POP enzyme and the capacity of the POP enzyme to process the substrates on solid-phase without the need of spacers on PEGA_1900_ and attaching them by the *C*-terminal end.

Our study demonstrates that the HMBA linker, which was used to connect the peptide with the solid support by an ester bond, cannot be used in this system. The design of such a peptide library will require the use of a distinct linker system that is compatible with the SPPS and the enzymatic assay. A test was also conducted with the PLL linker and showed its compatibility with the synthetic methodology and the enzymatic assay. This linker can therefore be considered for the future design of an OBOC library for the POP enzyme.

## Materials and Methods

### Reagents and instrumentation

Protected amino acids and other residues for SPPS were purchased from Iris Biotech GmbH, Anaspec Inc., Aldrich, Luxembourg Industries and Calbiochem-Novabiochem AG. Handles were supplied by Calbiochem-Novabiochem AG and Merck; and resins from Polymer Laboratories, Rapp Polymere GmbH, Calbiochem-Novabiochem AG, Matrix Innovation and CBL Patras. PyBOP and TBTU were supplied by Iris Biotech GmbH, DIEA was supplied by Merck and DIPCDI was obtained from Fluka. DMAP was acquired from Acros Organic, HOBt from S.D.S., Applied Biosystems supplied PyAOP and MeldalChemy S.L. the HOAt. Piperidine was acquired from SDS and α-ciano-4-hydroxycinnamic acid (ACH) from Aldrich. Fmoc-4-[4-(1-aminoethyl)-2-methoxy-5-nitrophenoxy]butyric acid was supplied by Iris Biotech GmbH.

Acetic anhydride, ninhydrin and phenylsilane were purchased from Fluka. Triethylamine was obtained from Alta Aesar and succinic anhydride from Merck. Sodium hydroxide was obtained from Panreac Quimica S.A. Monobasic potassium phosphate and dibasic sodium phosphate were purchased from Sigma-Aldrich.

Nylon filters of 0.45 µm and PVDF filters of 0.45 µ were obtained from Millipore. Syringes of 1 mL, 2 mL, 5 mL, 10 mL, 20 mL and 50 mL were purchased from BD Discardit II and BD Plastikpack. Falcons were obtained from Discardir TT and Deltalab, respectively. Solvents for peptide synthesis (DMF, DCM) and RP-HPLC (MeCN, MeOH) were obtained from SDS. Trifluoroacetic acid was supplied by Fluorochem. Ammonia was purchased from Aldrich, THF from SDS and acetone and ethanol from Panreac. Tert-butyl methyle oxide was obtained from SDS, hydrochloric acid from Scharlab and acetic acid from Riedel-de Haën.

Buffers for the activity assays were prepared by using a magnetic shaker from IKA, RCT basic and a pHmeter, Crison, MicropH 2002 - Crison 52-01. Two balances were used in the different stages of the study: Mettler Toledo, AB-S (0.1 mg) and PB-S (0.01 mg).

Centrifugation was carried out by a Beckmann Coulter, Allegra 21 R and a Heidolph Unimax 1010 shaker. A spectrophotometer UV-Vis Shimadzu, model UV mini-1240, was used to quantify the Fmoc deprotections. The micropipettes used were Gilson, Pipetman, P2, P20, P200, P1000. The water was obtained from a Milli-Q A10 system (Millipore).

The rotary evaporator was a Helidolph, Laborota 4003 with Vacuum pump Vacubrand, MZ 2C. Two incubators were used: Camlab Microtherm and Koxke. Finally, the lyophilizer was a Virtis 12EL and the speed-vac an eppendorf concentrator 5301. HPLC chromatograms were recorded on a Waters model *Alliance 2695* with Photodiode array detector 996 from Waters (Waters, Milford, USA) using a Symmetry C_18_ column (150×4.6 mm×5 µm, 100 Å, Waters) and a 717 Waters automatic injector, with a software *Millenium* version 4.0. Solvents were H_2_O (0.045% TFA) and MeCN (0.036% TFA), previously passed through a Millipore system with a filter of 0.45 µm, and samples were previously passed through a 0.45-µm filter. The working flow was 1 mL/min.

Some of the initial compounds were purified in an HPLC semi-preparative Waters 600 with Dual absorbance detector Waters 2487 (Waters, Milford, USA), with a Symmetry C_18_ column (100×30 mm×5 µm, 100 Å, Waters), which also incorporates a Waters 2700 automatic injector and a Waters Fraction Collector II. The system was controlled by the software *MassLynx 3.5*. Solvents were H_2_O (0.1% TFA) and MeCN (0.05% TFA), previously passed through a Millipore system with a filter of 0.45 µm, and samples were previously passed through a 0.45-µm filter. The working flow was 10 mL/min.

Mass spectra were recorded on a MALDI Voyager DE RP time-of-flight (TOF) spectrometer (PE Biosystems, Foster City, CA, USA), by using a N_2_ laser of 337 nm. Sample preparation consisted of mixing 1 µL of the sample with 1 µL of the MALDI matrix (ACH). Then, 1 µ L of this mix was dropped on the MALDI plate and dried by air. The matrix consisted of a solution of α-ciano-4-hydroxycinnamic acid (ACH) of 10 mg/mL in MeCN:H_2_O (1∶1) with 0.1% TFA.

The HPLC-MS consisted of a HPLC Waters model *Alliance 2796*, a Water 2700 automatic injector, UV/Vis Dual absorbance detector Waters 2487 and a ESI-MS model Micromass *ZQ*, with the software *Masslynx* version 4.0 (Waters, Milford, USA). This system used a Symmetry C_18_ column (150×3.9 mm×5 µm, 300 Å, Waters). Solvents were H_2_O (0.1% formic acid) and MeCN (0.07% formic acid), previously passed through a Millipore system with a filter of 0.45 µm, and samples were also previously passed through a 0.45 µm filter. The working flow was 1 mL/min.

Fluorescence was measured using a Jasco-J810 CD Spectropolarimeter. For these measures, the system incorporated a JASCO Peltier for the control of the temperature and a monochromator FMO-427 for the measurement.

After optimizing the synthetic methodology, the last syntheses were conducted using the automatic synthesized 433A, Applied Biosystems.

HPLC chromatograms for the amino acids analysis were recorded on a Waters model *600 Controller* with Photodiode array detector 2487 (Waters, Milford, USA) and a 717 plus Waters automatic injector, with the software *Millenium* version 4.0.

### General protocol for solid-phase synthesis

In all cases the synthesis was conducted with L-amino acids. Mostly, solid-phase peptide elongation and other solid-phase manipulations were done manually in polypropylene syringes, each fitted with a polyethylene porous disk. However, after optimizing the synthetic methodology, analogs were synthesized automatically. Solvents and soluble reagents were removed by suction. Washings between synthetic steps were carried out with DMF (5×1 min) and DCM (5×1 min) using 5 mL of solvent/g resin each time. During couplings the mixture was allowed to react with intermittent manual stirring and in some cases mixing was conducted using a shaker.

### SPPS using Fmoc/^t^Bu strategy

Synthesis of *C*-terminal amide peptides was carried out by using Sieber amide resin (0.71 mmol/g.). Peptides with an acid group at the *C*-terminal side were synthesized using 2-Chlorotrityl resin, which presented an initial substitution of 1.5 mmol/g. This substitution was reduced by the partial incorporation of the first protected amino acid until 0.5–0.7 mmol/g. Finally, anchored peptides or re-anchoring of the peptides were produced in PEGA_1900_ resin (0.2 mmol/g).

All the peptides were synthesized using the Fmoc/^t^Bu strategy, which allows the incorporation of amino acids with protected lateral chains in a future OBOC peptide library.

### Attachment of the first amino acid to 2-Chlorotrityl resin

The first amino acid coupling over the 2-Chlorotrityl resin was carried out by adding 0.5 eq. of Fmoc-amino acid and 10 eq. of DIEA in DCM, and mixing during 80 min. The non-reacted positions were capped with MeOH (1 mL/g. resin) by mixing during 10 min. After washing the resin with DCM (5×1 min) and DMF (5×1 min), the Fmoc group was removed by piperidine 20% DMF and quantified, which allowed the coupling quantification.

### Attachment of the first amino acid to Sieber resin

Sieber resin is commercially sold in Fmoc-protected form. Removal of the Fmoc group was carried out after swelling the resin in DCM (5×1 min) and DMF (5×1 min) by treatment with 20% piperidine in DMF (1×1 min followed by 2×10 min). After washing the resin with DCM (5×1 min) and DMF (5×1 min), the Ninhydrin or Kaiser test [24] was carried out to ensure the deprotection of the resin.

### Initial conditioning of the PEGA _1900_ resin

The PEGA_1900_ resin was conditioned by solvating in MeOH (5 min.), washing with DCM (10×1 min) followed by acid treatment (DCM with TFA 1%, 10×1 min) and neutralization (DCM with DIEA 5%, 10×1 min). Finally the resin was washed with DCM (10×1 min) and DMF (10×1 min).

### Removal of the Fmoc ((9-fluorenylmethyl) carbamate) group

Fmoc group was removed after swelling the resin in DMF by three treatments of the resin with 20% piperidine in DMF (3–4 mL/g resin): the first of 1 min and the last two of 10 min. The resin was then washed with DMF (10×1 min) and DCM (10×1 min).

### Removal of the Boc (tert-Butoxycarbonyl) group

Boc group was removed manually by repeated treatments of the resin with TFA 55%(v/v) in DCM. In many cases, this step was conducted at the same time that the peptide was cleaved from the Sieber resin because only a 2% TFA in DCM was necessary for the cleavage.

### Removal of the Alloc (allyloxycarbonyl carbamate) group

Removal of the Alloc-protecting group was achieved under argon atmosphere in anhydride CH_2_Cl_2_ in the presence of the scavenger phenylsilane (10 eq.) with Pd(PPh_3_)_4_ (0.1 eq.) (2×15 min). Next, the resin was washed with DCM (5×1 min) and DMF (5×1 min), followed by a solution of sodium diethylditiocarbamate 0.02 M in DMF (2×30 min) to remove the remaining palladium.

### Coupling methodology

The general procedure for the coupling of a protected amino acid (3 or 4 eq.) was the following: α-protected amino acid (3 eq. or 4 eq.), TBTU (3 eq. or 4 eq.) with DIEA (6 or 8 eq.) in DMF (1–3 mL/g resin). The mixture was left to react with intermittent manual stirring for 75–90 min. Finally, the solvent was removed by filtration, and the resin was washed by using DMF (10×1 min) and DCM (10×1 min). The extent of the coupling was checked by using the Ninhydrin [24], Chloranil [25] or De Clercq [26] tests (depending on the nature of the amino acid group).

Special couplings were conducted for different residues, as it is following described. Coupling onto proline residue was always carried out by PyBOP/HOAt/DIEA (3∶3∶9∶9 equiv. or 4∶4∶12∶12 equiv.) in DMF. Coupling of Abz was carried out by using Fmoc-Abz-OH/PyBOP/HOAt/DIEA (4∶4∶12∶12 equiv.) in DMF. Coupling onto the O_2_Oc spacer was conducted by using Fmoc-O_2_Oc-OH/PyAOP/DIEA (4∶12∶12 equiv.) in DMF. The coupling of the first amino acid onto the HMBA linker attached to PEGA resin was carried out by 1-mesityl-sulphonyl-3-nitro-1,2,4-triazole (MSNT) in the presence of *N*-Methyl imidazole (MeIm) and the Fmoc-AA-OH in argon atmosphere (2.5∶2.35∶2 equiv.) [14].

### Coupling of the linkers to PEGA_1900_ resin

The method used for the incorporation of the handle HMBA to the PEGA was to dissolve the handle HMBA (3 eq.) in DMF/DCM. Next, TBTU/DIEA (2.85∶6 equiv.) were added and mixed for 3 min. The mixture was added to PEGA resin (already conditioned) and was kept at room temperature for 4 h with gentle shaking. Although, the method describe by Meldal [19] included two couplings, the coupling yield was increased with an additional third coupling. After washing with DMF followed by DCM, the Nynhydrin test [24] and the alcohol test [25] were conducted to ensure the good coupling.

In the synthesis of the *N*-terminal anchored peptides, after the HMBA linker, the succinic anhydride was also coupled by using DIEA (2×1 h). For the optimization of the anchoring protocol the *N*-1-Fmoc-1,5-diaminopentane-HCl was used as a simulation of the future peptide, which would allow the quantification of the coupling methodology based on the removal of the Fmoc group. The peptides were coupled by using PyBOP/HOAt/DIEA (5∶5∶7.5 equiv.)

### Cleavage from the resin and work-up

After the last residue coupling and control test, the resin was washed with DMF (5×1 min.) and DCM (5×1 min). Next, the control peptides were cleaved by using a cleavage cocktail (2 mL/100 µmoles of peptide), which depended on the resin and the remaining protecting groups. Cleavage of the control peptides from Sieber amide resin was accomplished by using 3% TFA in DCM (5×10 min) [22]. While, cleavage of the control peptides (without protecting groups) from 2-Chlorotrityl resin was accomplished by using 2% TFA in DCM (5×10 min).

Cleavage of the control peptides (containing the Boc-protecting group) from Sieber amide resin was accomplished by treating the resin with 55% TFA in DCM. After the cleavage, the filtrates were collected and evaporated under N_2_ and work-up with *tert*-butylmethylether anhydrous. After centrifugation at 4°C and 4000 rpm during 5 min and removal of the supernatant, the peptide was solved in H_2_O/MeCN (1∶1) and lyophilized. Finally, the peptides were characterized by HPLC and MALDI-TOF Spectrometry.

### Cleavage of the peptides from PEGA_1900_ resin with HMBA linker

After the synthesis, a control cleavage of the analogs attached by the *C*-terminal and the *N*-terminal to PEGA resin was conducted in order to analyze them by HPLC and MALDI-TOF. In this case, the cleavage cocktail was 0.01 N NaOH and cleavage was carried out for 2 h with automatic shaking [19]. The mixture was filtered and the solution was neutralized with 0.01 N HCl and lyophilized. The peptide was solved in H_2_O/MeCN (1∶1) and analyzed by HPLC and MALDI-TOF.

After the enzymatic assay, the positive beads were placed on the MALDI plate and the peptide was cleaved using ammonia/THF vapor for 24 h. Then, 1 µL of MeCN/H_2_O 1∶1, with 0.5% TFA) was added to each spot for cleaning. After evaporation, each spot was treated with 1 µL of AcOH/H_2_O/TFA (3∶4∶3), and the solvent was evaporated again. Finally, the MALDI matrix, consisting of a solution of MeCN/H_2_O/TFA/α-cyano-4-hydroxycinnamic acid (2 mg/mL) (49.5∶49.5∶0.5∶0.5 equiv.), was deposited on each spot and the plate was then analyzed by MALDI-TOF Spectrometry [24].

### Cleavage of the peptides from the photolabile linker

Photocleavage of the peptides attached to the resin using the photolabile peptide was carried out by using a UV lamp of 230 V, 50 Hz at a wavelength of 365 nm during 3 h.

### Peptide purification

Some of the peptides were purified by reverse phase HPLC at semi-preparative scale. The purification conditions were first optimized by the RP-HPLC at analytical scale. The crude product was purified using a Symmetry C_18_ column (100×30 mm×5 µm, 100 Å, Waters) with a flow of 10 mL/min and the detection was carried out at 220 nm. The collected fractions were analyzed by HPLC at analytical scale and the pure ones were mixed and lyophilized.

### Study of the resins

To study the effect of the distinct solvents, the swelling capacity of the five amino functionalized resins were measured by swelling the resins with diverse solvents ordered by increasing polarity: DCM, DMF, MeOH, water and Na^+^/K^+^ phosphate buffer with POP enzyme. The degree of swelling was measured by weighing a known amount (50 mg) of each resin in a syringe. Each resin was then swollen in DMF (10×1 min), incubated with that solvent for 5 min and the solvent was then removed without suction. Finally, the volume was measured. These operations were repeated for each solvent and the swelling in mL/g was calculated.

To determine the bead size and homogeneity of the resins, the five resins were swelled in Na^+^/K^+^ phosphate buffer 100 mM with the POP enzyme and optical microscopy images were taken using an Optical Microscope Nikon Eclipse TS 100 and a Digital camera Cooled MDC Nikon. The diameter of 100 beads from each kind of resin were measured and treated statistically. To determine whether the resins were auto fluorescent, the five resins were swelled in Na^+^/K^+^ phosphate buffer 100 mM with the POP enzyme and were analyzed by fluorescence microscopy Leica DMRB with distinct light sources to ensure that the bead itself was not auto fluorescent.

### Conditions for the activity assay in solution

The conditions for the activity assays were 100 mM of Na^+^/K^+^ phosphate buffer pH 8.0, at several POP concentrations (from 0.5 ngr/mL to 1.35 ngr/mL) and substrate concentrations of 50 or 90 µM. The incubation was carried out during 1 h at 37°C and the reaction was stopped by heating at 95°C for 5 min.

Human POP was obtained by expression in *E. coli*, and subsequent affinity purification using a His tail fusion [7]. POP stock solutions were prepared at a concentration of 200 ngr/µL in Tris HCl 50 mM pH 8. Stock solutions for the substrate were prepared in H_2_O : MeCN (1∶1, v/v) for each peptide, and the activity buffer was prepared with Na_2_HPO_4_ and KH_2_PO_4_ until pH 8 was reached.

### Conditions for the activity assay in solid-phase

The conditions for the activity assays were 100 mM of Na^+^/K^+^ phosphate buffer pH 8.0, at several POP concentrations (from 0.5 ngr/mL to 1.35 ngr/mL) and substrate concentrations of 300 µM. The incubation was carried out for 10 min, 20 min, 30 min, 1 h, 2 h, and 3 h at 37°C and the reaction was stopped by centrifugation (separating the solution and the solid phase).

### Activity assay and analysis of the analogs in the presence of a POP inhibitor

In this experiment, to study the effect of POP and the POP media, Z-pro-prolinal inhibitor (ZPP) was used to inhibit the enzyme. ZPP is a commonly used reversible and potent inhibitor [7]_._ ZPP was purchased from Biomol Research laboratories Inc. and dissolved in DMSO at 100 mM. To ensure the inhibition of the enzyme, 10 eq. of the inhibitor versus the POP enzyme were used (concentrations were previously tested in other systems). After pre-incubating the POP with 20 eq. of inhibitor during 15 min, the substrate was added and incubated for 30 min at 37°C. The reaction was stopped by centrifugation or heating depending on the substrate, and MeCN was added to the samples, which were centrifuged and lyophilized. Finally, the samples were dissolved in H_2_O/MeCN and analyzed by HPLC-UV spectrum.

### Optical microscopy measurements of resin beads

The size homogeneity of the resin beads was determined by taking optical microscopy images with a Nikon Eclipse TS 100 optical microscope and a Digital camera Cooled MDC Nikon, and measuring the diameter of the beads.

### Fluorescence microscopy images and cleavage of the peptides from PEGA_1900_ resin containing the photolabile linker

Microscopy fluorescence images of the resin and the peptides anchored to the resin were carried out with a fluorescence microscopy Leica DMRB using a Hg vapor lamp and a stereoscopic fluorescence microscope Leica MZFLIII. The images were taken with a Digital camera Cooled CCD Micromax, with RTE 782-Y format, 782×580 pixels, cooled until −15°C by a system of pelters and using the excitation filter of 360/40 nm and emission filter of 420/40 nm.

## References

[1] Maes M, Goossens F, Scharpe S, Calabrese J, Desnyder R (1995). Alterations in plasma prolyl endopeptidase activity in depression, mania and schizophrenia: effects of antidepressants, mood stabilizers, and antipsychotic drugs.. Psychiatry Res.

[2] García-Horsman J, Männisto P, Venäläinen J (2007). On the role of prolyl oligopeptidase in health and disease.. Neuropeptides.

[3] Nanteuli D, Portevin B, Lepagnol L (1998). Prolyl Endopeptidase inhibitors: a new class of memory enhancing drugs.. Drugs of the future.

[4] Fulop V, Bocskei Z, Polgar L (1998). Prolyl oligopeptidase: an unusual *β*-propeller domain regulates proteolysis.. Cell.

[5] Tarragó T, Sabidó E, Kogan M, De Oliviera E, Giralt E (2005). Primary structure, recombinant expression and homology modelling of human brain prolyl oligopeptidase, an important therapeutic target in the treatment of neuropsychiatric diseases.. J Peptide Sci.

[6] Tarragó T, Kichik N, Seguí J, Giralt E (2007). The natural product berberine is a human Prolyl Oligopeptidase Inhibitor.. Chem Med Chem.

[7] Tarragó T, Frutos S, Rodriguez-Mias R, Giralt E (2006). Identification by ^19^FNMR of traditional Chinese Medicinal Plants Possessing Prolyl Oligopeptidase Inhibitory Activity.. Chem Bio Chem.

[8] Fülöp V, Zoltan S, Renner V (2001). Structures of Prolyl Oligopeptidase Substrate/Inhibitor Complexes.. J Biol Chem.

[9] Lam KS, Lebl M, Krchnak V (1997). The “One-Bead One-Compound” Combinatorial Library methods.. Chem Rev.

[10] Gershkovich A, Kholodovich V (1996). Fluorogenic substrates for proteases based on intramolecular fluorescence energy transfer (IFETS).. Journal of Biochemical and Biophysical Methods.

[11] Leon S, Quarrell R, Lowe G (1998). Evaluation of resins for on-bead screening: a study of papain and chymotrypsin specificity using Pega-Bound combinatorial peptide libraries.. Bioorganic & Medicinal Chemistry Letters.

[12] Sauerbrei B, Jungmann V, Waldmann H (1998). An enzyme-labile linker group for organic synthesis on solid supports.. Angew Chem Int Ed.

[13] Kress J, Zanaletti R, Amour A, Ladlow M, Frey J (2002). Enzyme Accessibility and Solid Supports: Which Molecular Weight Enzymes Can Be Used on Solid Supports? An Investigation Using Confocal Raman Microscopy.. Chem Eur J.

[14] Meldal M (2005). Smart Combinatorial Assays for the Determination of Protease Activity and Inhibition.. QSAR Comb Sci.

[15] Halling PJ (2006). Understanding enzyme action at solid surface.. Biochemical Society Transactions.

[16] Pastor J, Granados G, Carulla N, Rabanal F, Giralt E (2007). Redesign of Protein domains using One-Bead One-Compound Combinatorial Chemistry.. J Am Chem Soc.

[17] Farrera-Sinfreu J, Royo M, Albericio F (2002). Undesired removal of the Fmoc group by the free ε-amino function of a lysine residue.. Tetrahedron letters.

[18] Yao N, Wu CY, Xiao W, Lam K (2008). Discovery of High-Affinity Peptide Ligands for Vancomycin.. Peptide Science.

[19] Renil M, Ferreras M, Delaisse J, Coged N, Meldal M (1998). PEGA supports for combinatorial peptide synthesis and solid-phase enzymatic library assays.. J Peptide Sci.

[20] Letsinger R, Kornet M (1963). Popcorn polymers as a support in multistep syntheses.. Journal of American Chemical Society.

[21] Felix A, Merrifield R (1970). Azide Solid Phase Peptide Synthesis.. J Am Chem Soc.

[22] Léger R, Yen R, She M, Lee V, Hecker S (1998). N-linked Solid Phase Peptide Synthesis.. Tetrahedron Letters.

[23] Shin DS, Kim KG, Kim EM, Kim M, Park HY (2008). Solid-Phase Peptide Library Synthesis on HiCore Resin for Screening Substrate Specificity of Brk Protein Tyrosine Kinase.. J Comb Chem.

[24] Kaiser E, Colescott R, Bossigner C, Cook P (1970). Color test for detection of free terminal amino groups in solid-phase synthesis.. Analytical Biochemistry.

[25] Vázquez J, Qushair G, Albericio F (2003). Qualitative Colorimetric Tests for Solid Phase Synthesis.. Methods in enzymology.

[26] Madder A, Farcy N, Hosten NGC, De Muynck H, De Clerq J (1999). A novel Sensitive Colorimetric Assay for Visual Detection of solid-phase-bound amines.. European Journal of Organic Chemistry.

[27] Marani M, Paradís-Bas M, Tulla-Puche J, Côté S, Camperi S (2007). From One-Bead One-Compound Concept to One-Bead One-Reactor.. J Comb Chem.

